# Evaluation of the oxidative stress alleviation in *Lupinus albus* var. orden Dorado by the inoculation of four plant growth-promoting bacteria and their mixtures in mercury-polluted soils

**DOI:** 10.3389/fmicb.2022.907557

**Published:** 2022-09-29

**Authors:** Daniel González-Reguero, Marina Robas-Mora, Agustín Probanza, Pedro A. Jiménez

**Affiliations:** Department of Pharmaceutical Science and Health, San Pablo University, CEU Universities, Boadilla del Monte, Spain

**Keywords:** heavy metal, reactive oxygen species (ROS), catalase (CAT), superoxide dismutase (SOD), ascorbate peroxidase (APX), glutathione reductase (GR), phytoprotection

## Abstract

Mercury (Hg) pollution is a serious environmental and public health problem. Hg has the ability to biomagnify through the trophic chain and generate various pathologies in humans. The exposure of plants to Hg affects normal plant growth and its stress levels, producing oxidative cell damage. Root inoculation with plant growth-promoting bacteria (PGPB) can help reduce the absorption of Hg, minimizing the harmful effects of this metal in the plant. This study evaluates the phytoprotective capacity of four bacterial strains selected for their PGPB capabilities, quantified by the calculation of the biomercuroremediator suitability index (IIBMR), and their consortia, in the *Lupinus albus* var. orden Dorado. The oxidative stress modulating capacity in the inoculated plant was analyzed by measuring the activity of the enzymes catalase (CAT), superoxide dismutase (SOD), ascorbate peroxidase (APX), and glutathione reductase (GR). In turn, the phytoprotective capacity of these PGPBs against the bioaccumulation of Hg was studied in plants grown in soils highly contaminated by Hg vs. soils in the absence of Hg contamination. The results of the oxidative stress alleviation and Hg bioaccumulation were compared with the biometric data of *Lupinus albus* var. orden Dorado previously obtained under the same soil conditions of Hg concentration. The results show that the biological behavior of plants (biometrics, bioaccumulation of Hg, and activity of regulatory enzymes of reactive oxygen species [ROS]) is significantly improved by the inoculation of strains B1 (*Pseudomonas moraviensis*) and B2 (*Pseudomonas baetica*), as well as their corresponding consortium (CS5). In light of the conclusions of this work, the use of these strains, as well as their consortium, is postulated as good candidates for their subsequent use in phytostimulation and phytoprotection processes in areas contaminated with Hg.

## Introduction

Heavy metal pollution is an environmental threat that affects all types of living organisms, including plants, animals, and humans. Particularly, mercury (Hg) is one of the most polluting heavy metals. Even at relatively low concentrations, it has the ability to bioaccumulate and transmit through the food chain (Bjørklund et al., 2019). The accumulation of Hg can lead to pathologies that affect the central nervous system, one of the most important being Minamata syndrome (Gil-Hernández et al., 2020; Marumoto et al., 2020).

The presence of Hg at low concentrations is widely described in numerous ecosystems. Exceptionally, environments with extremely high concentrations of this heavy metal have also been described, such as those detected in the mining region of Almadén (>8889 μg/g de Hg) (US Environmental Protection Agency, 2011). The presence of this heavy metal and other polluting substances can affect plant development (Kim et al., 2017; Loix et al., 2017; Sachdev et al., 2021).

One way to evaluate the effects of Hg pollution on plant development is by studying its response to this abiotic stress. To do this, plants synthesize antioxidant enzymes that fight reactive forms of oxygen (ROS). ROS accumulation alters the metabolic balance and physiology of the plant. The main types of ROS are hydrogen peroxide (H_2_O_2_), hydroxyl radicals (−HO), oxygen singlet (^1^O_2_), and superoxide anion (O^–^_2_). Its cytoplasmic accumulation induces high oxidative stress and can produce harmful effects on the cell. To mitigate these effects, detoxifying mechanisms are expressed. However, when antioxidant processes and detoxification mechanisms are not able to eliminate excess ROS, oxidative stress harms the plant (Loix et al., 2017). It is proven that Hg induces oxidative stress causing lipid peroxidation, enzymatic inactivation, DNA and membrane damage (Cargnelutti et al., 2006; Tamizselvi and Napoleon, 2022), inhibits photosynthesis, transpiration, and nutrient transport in plants (Cargnelutti et al., 2006; Zhou et al., 2007; Ajitha et al., 2021). Its effects can even lead to the premature death of the plant (Ercal et al., 2001). The accumulation of these reactive species in cells can be reduced by activating different enzyme systems, including catalase activities (CAT), superoxide dismutase (SOD), ascorbate peroxidase (APX), and glutathione reductase (GR) (Loix et al., 2017; Sachdev et al., 2021).

The use of plant growth-promoting bacteria (PGPB) in soils contaminated with Hg has traditionally focused on the phytoextraction of this metal, as well as on the direct promotion of plant growth (Gontia-Mishra et al., 2016; Mariano et al., 2020; González et al., 2021a). These bacteria have also been used to improve the resistance of plants against different situations of abiotic stress such as salinity or desiccation (Ansari et al., 2021; Ha-Tran et al., 2021; Khalilpour et al., 2021; Ali et al., 2022), as well as the oxidative stress produced by Hg (Cho and Park, 2000; Cargnelutti et al., 2006; Ajitha et al., 2021; Quiñones et al., 2021; Çavuşoğlu et al., 2022). To alleviate the harmful effect of pollutants, plants rely heavily on bacteria present in their rhizospheres.

The present work studies the effect of the inoculation of four PGPB strains and their combination in consortia formed by pairs, on the oxidative stress of *Lupinus albus* var. orden Dorado grown in different growing matrixes with the presence of Hg. Likewise, the phytoprotective effect of PGPBs that manifest the best results in the reduction of oxidative stress is studied. As an indicator, we use the concentration of Hg accumulated in plants. Additionally, we relate these variables to biometrics and the activity of ROS-regulating enzymes.

## Materials and methods

### Bacterial strains and mixtures

The isolates used in this study come from the free soil and rhizosphere of plants that grow naturally on plot 6 of the mining district of Almadén in Ciudad Real, Spain (Millán et al., 2007). The strains were selected based on their Biomercuroremediator Suitability Index values (BRMSI) (Robas et al., 2021), which evaluates PGPB activities and their tolerance to Hg. The tolerance to Hg is assessed using the minimum bactericidal concentration (MBC) and the PGP activities are as follows: production of auxin (3-indoleacetic acid: IAA), presence of the enzyme 1-animociclopropane-1-carboxylate decarboxylase (ACCd), production of siderophores (SIDs), and the solubilizing capacity of phosphates. The BMRSI is calculated using the following formula, where 1 and 0 for the ACCd and PO_4_^–3^ indicate presence or absence:

BRMSI = [IAA (μg mL^–1^) + ACCd (1/0) + SID (cm) + PO_4_^–3^ (1/0)] + [MBC Hg (μg mL^–1^)]

The PGPB capacity in the presence of Hg of the four bacterial isolates (Table 1) was analyzed by González et al. (2021b) (BRMSI Supplementary Table 1). The activity of the four strains was tested, as well as the combination consortium in pairs (Table 2).

**TABLE 1:** Bacterial isolates according to their BMRSI in the presence of Hg (González et al., 2021b).

Strain	HgCl_2_ tolerance (μg/mL)	BMRSI	Strain origin	16S rRNA identification
A1	140	6.54	*Avena sativa*	*Brevibacterium frigoritolerans*
A2	140	7.30	*BS*	*Bacillus toyonensis*
B1	140	7.20	*BS*	*Pseudomonas moraviensis*
B2	140	6.92	*Avena sativa*	*Pseudomonas baetica*

**TABLE 2:** Consortia formed to screen the strains in Table 1.

	CS1	CS2	CS3	CS4	CS5	CS6
Strains	A1 + B1	A1 + A2	A1 + B2	B1 + A2	B1 + B2	A2 + B2

The results of the biometrics of *Lupinus albus* var. orden Dorado inoculated with these PGPB and their respective consortia are shown in Supplementary Table 2. In all the experiments carried out, “control” means without inoculum.

The four bacteria isolates were subjected to the mutual compatibility test by cross streak method (Supplementary Figure 1) in standard method agar plates (SMA, Pronadisa ^®^, Madrid, Spain). No inhibition was observed on the cross point in any of the combinations, which indicate the compatibility among the isolates.

### Tested plants

*Lupinus albus* var. orden Dorado seeds were used from the seed bank of the Technological and Scientific Research Centre of Extremadura.

### Growing matrixes

Four types of growing matrixes were used: to free soil from the mining district of Almadén and sterile vermiculite. The characteristics of the different growing matrixes are as follows:

• Contaminated soil, high concentration of Hg (“Soil +Hg”), from “Plot 6” of the mining district of Almadén (Table 3).
• Control soil with low Hg concentration (“Soil −Hg”), obtained from “Plot 2” of the mining district of Almadén. The concentration of soluble and interchangeable Hg in this plot is low enough to be considered negligible (Table 3).
• Vermiculite without Hg (“Vermiculite −Hg”): vermiculite is an inert substrate with neutral pH commonly used in hydroponic crops.
• Vermiculite was added with a solution of 8 mg/kg of HgCl_2_ (concentration of Hg analogous to that found in the soluble fraction of the plot “Plot 6”) (“Vermiculite +Hg”).

**TABLE 3:** Hg speciation on study soils (Millán et al., 2007).

Soil	Total Hg (mg/Kg)	Soluble Hg (mg/Kg)	Exchangeable Hg (mg/Kg)
Plot 6 (Soil +Hg)	1710	0.609	7.3
Plot 2 (Soil −Hg)	5.03	0.0417	0.285

### Seed pre-germination

As a preliminary step, the seeds were soaked in water at 4°C for 24 h. The surface was sterilized with three washes of 70% ethanol for 30 s (Abdel Latef et al., 2017). Trays were used with sterile vermiculite and watered with sterile water to field capacity. The seeds were then sown and kept in darkness for 72 h at 25°C. Seeds with an emerged radicle of 3 ± 0.2 cm were selected for the study.

### Sowing conditions and inoculation with the strains and mixtures

Sterile forest trays were used (Plásticos Solanas S.L., Zaragoza, España), each of them composed of 12 alveoli of 18 cm in height, with a capacity of 300 cm^3^, and a light of 5.3 cm × 5.3 cm. Eleven trays were used for each type of growing matrix. To avoid cross-contamination, four pre-germinated seeds were sown in each alveolus. In each tray, a single bacterial strain (or consortium) and/or control was inoculated, in such a way that 48 seeds were tested for each condition.

A bacterial suspension in 0.45% saline was performed and the inoculum density was adjusted to 0.5 McFarland. Each seed was inoculated with 1 ml of the suspension. To the control, seeds were added to 1 ml of 0.45% saline per seed without bacterial suspension.

### Plant growth conditions

A plant growth chamber (phytotron) equipped with white and yellow light with a photoperiod of 11 h of light was used (light intensity: 505 μmol m^–2^ s^–1^, temperature stable at 25 ± 3°C). Irrigation was carried out every 48 h by capillarity with sterile water, with an experimental volume of 350 mL/tray (12 alveoli).

### Harvest

Twenty-one days after seeding, the plants were harvested. To carry out the enzymatic measurements, six replicas were used for each treatment. Each replica was formed by a *mixture* of two plants (one plant per alveolus) until reaching 3 g. Four enzymatic measures related to protection against oxidative stress in plants were performed. The enzymatic activities tested were superoxide dismutase (SOD), catalase (CAT), ascorbate peroxidase (APX), and glutathione reductase (GR).

To study the concentration of accumulated Hg, three replicates were taken per treatment of each growing matrix. Each sample consists of 12 plants (three plants per alveolus) up to 25 g per sample. The analysis was only carried out in those treatments with greater statistical significance.

### Antioxidative defense enzymes

The enzymes were extracted at 4°C starting from 1 g of fresh sample per replica, with a mortar and using 50 mg polyvinylpolypyrrolidone (PVPP) and 10 ml of the following medium: 50 mM of K-phosphate buffer (pH 7.8) with 0.1 mM EDTA (for SOD, CAT, and APX). The same medium, supplemented with 10 Mm of β-mercaptoethanol was used for GR.

#### Superoxide dismutase activity

The SOD activity was measured based on the ability of SOD to inhibit the reduction of tetrazoyl nitro-blue (NBT) by photochemically generated superoxide radicals. A SOD unit is defined as the amount of enzyme needed to inhibit the NBT reduction rate by 50% at 25°C (Burd et al., 2000).

#### Catalase activity

The method of Aebi (1984) was carried out. H_2_O_2_ consumption was monitored for 1 min at 240 nm. This was carried out by mixing 50 mM potassium phosphate buffer with 10 mM of H_2_O_2_ and 100 μL of the extract.

#### Ascorbate peroxidase activity

The reaction was measured in a total volume of 1 mL that contains 80 nM of potassium phosphate buffer, 2.5 mM H_2_O_2_, and 1M sodium ascorbate. To determine the oxidation ratio of ascorbate, H_2_O_2_ was added to begin the reaction and the reduction of absorbances was measured for 1min at 290 nm (Amako et al., 1994).

#### Glutathione reductase activity

Glutathione reductase activity was estimated spectrophotometrically, according to the method of Carlberg and Mannervik (1985) at 25°C and 340 nm. The reaction mixture contained 50 mM of buffer Tris–MgCl_2_, 3 mM, 1 mM of GSSG, 50 μl of enzyme, and 0.3 mM NADPH, which were added to initiate the reaction. The activity was calculated with the initial rate of the reaction and the molar extinction coefficient of NADPH (ε_340_ = 6.22 mM−1 cm^–1^).

### Analysis of Hg content in plant

The root and aerial fraction of each replica was dried in dry heat furnaces at 60°C for 24 h. It was sprayed and each fraction was digested separately in the acidic medium (HNO_3_/HCl 2/0.5% weight/volume) under pressure for the determination of trace elements according to the regulations UNE-EN 13805. The digest was analyzed by mass spectrometry with inductively coupled plasma (ICP-MS).

By using a calibration curve, a relationship between the concentration of the pattern (μg L^–1^ or mg L^–1^) and signal (ICP-MS) was established for each of the elements. The value of the element signal in the 12 samples is interpolated on the calibration line resulting in the total concentration of the element in the sample.

The values of the Hg pattern to establish the calibration line were as follows, expressed in μg/L: 0.00; 0.05; 0.10; 0.50; 1.00; 5.00; 10.00. Expression in mg kg^–1^ from μg L^–1^:


$$C\,f\,\left(\frac{\mu \,g}{K\,g}\right)=X\,\left(\frac{\mu \,g}{L}\right)\cdot D\cdot \frac{V\,\left(m\,L\right)}{W\,\left(g\right)}\cdot 10^{-3}$$

where Cf (mg kg^–1^) is the sample metal content, X (μg L^–1^) corresponds to the interpolated experimental value or the experimental value extrapolated from the standard addition, D is the dilution performed for determination, dilution factor, V (mL) corresponds to the flask volume, and W(g) to the sample weight.

### Statistical analysis

For statistical analysis, SPSS v.27.0 software was used (Version 27.0 IBM Corp, Armonk, NY, USA). The Kolmogorov–Smirnov test was performed to check the normality of all variables. Subsequently, an ANOVA of a Kruskal–Wallis factor was performed. For the statistical analysis of the total Hg concentration accumulated in the plant, the normality of the sample data was verified using the Shapiro–Wilk test. An ANOVA was performed to determine the existence of significant differences (*p*-value ≤ 0.05). Next, a *post hoc* analysis of less significance differences (LSDs) was performed with the aim of evaluating whether the differences in Hg concentration in the plant are significant. “Substrate with Hg” is considered to be the joint analysis of the data of vermiculite supplemented with Hg and soil with a high concentration of Hg. The joint analysis of the data for vermiculite without Hg and soil without Hg is considered “substrate without Hg.”

A principal component analysis (PCA) was performed starting with the 3D projection of the load factors. Next, an analysis was elaborated with the biometric data (Supplementary Table 2; González et al., 2021a), the concentration of Hg in the plant, and the results of the ROS enzymatic activity. All the statistical differences refer to the comparison of the variables that the plants manifest according to their inocula against their respective non-inoculated controls.

## Results

### Antioxidative defense enzymes analysis

Kruskal–Wallis ANOVA revealed that plants grown with the different inocula in the substrates without Hg showed no significant differences in the enzyme activity produced in response to oxidative stress. In contrast, in plants inoculated with strains B1 (*Pseudomonas moraviensis*) and B2 (*Pseudomonas baetica*), as well as their respective CS5 consortium, the differences in the activity of the four enzymes were significantly lower (*p*-value ≤ 0.001) when they were grown in soils with high levels of Hg.

**FIGURE 1:**
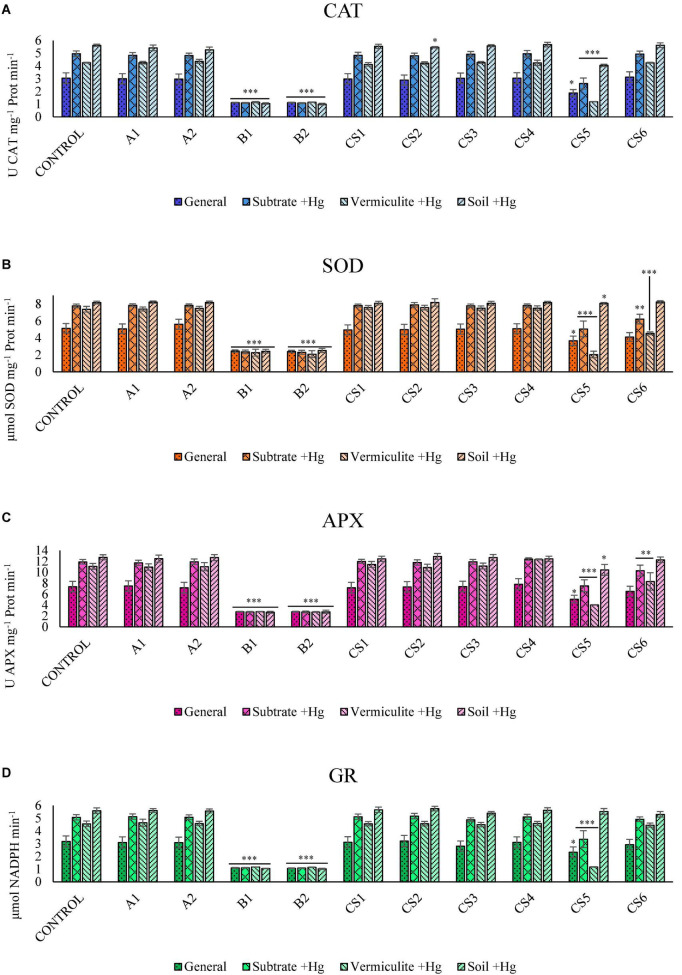
Kruskal–Wallis ANOVA results for enzyme activity: CAT **(A)**, SOD **(B)**, APX **(C)**, and GR **(D)**. Data clusters for statistical treatment: “General”: dataset for plants grown in all growing matrixes; “Substrates +Hg”: dataset for plants in Hg supplemented vermiculite (“Vermiculite +Hg”) and soil with high Hg concentration (“Soil +Hg”); “Vermiculite +Hg”: dataset for plants in supplemented vermiculite; “Soil +Hg”: dataset of plants in soil with Hg high concentration. The bars indicate the standard error. Asterisks indicate the level of significance compared to control; *p-value ≤ 0.05, ^**^p-value ≤ 0.003, and ^***^p-value ≤ 0.001.

Figure 1 shows the Kruskal–Wallis analysis and the comparison of means of the enzymes CAT (Figure 1A), SOD (Figure 1B), APX (Figure 1C), and GR (Figure 1D). Figures 1A–D shows the behavior of the activity of ROS-regulating enzymes of strains B1, B2, and their respective consortium (CS5). The CS6 consortium (formed by strains A2 and B2) is able to induce a significant reduction in the activity of the SOD enzyme by jointly analyzing substrates with high Hg concentration (Figure 1B).

**FIGURE 2:**
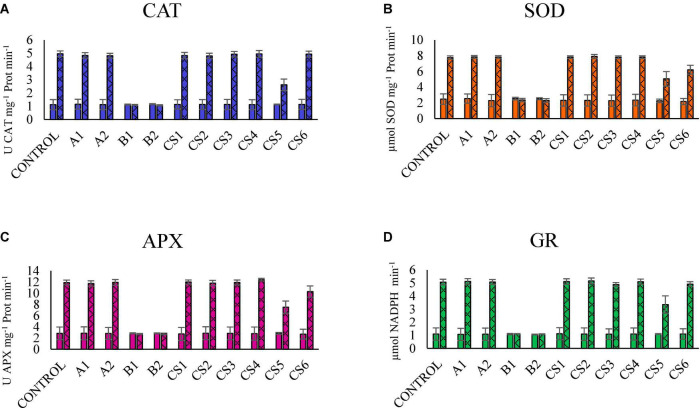
Comparison of the results of the enzymatic activity of CAT **(A)**, SOD **(B)**, APX **(C)**, and GR **(D)** in plants grown in the substrate without Hg (smooth) vs. with a high concentration of Hg (double scratching). The bars indicate the standard deviation.

Figure 2 shows the results of the enzymatic activities of plants subjected to different bacterial inoculums, comparing the behavior in the presence of Hg vs. the absence of Hg. We can observe that the reduction of the activity of the four enzymes in plants inoculated with strains B1 and B2 in soils with Hg reduces their activity to levels similar to those observed in plants grown in substrates in the absence of Hg.

### Analysis of Hg content in plant

In order to understand the bioaccumulation of Hg, we proceeded to analyze the samples of plants grown in soils with Hg whose inoculation induced a significantly lower enzymatic activity. Likewise, the data of plants inoculated with the same PGPB and grown in soils in the absence of Hg are collected comparatively (Table 4). The ability of *Lupinus albus to* bioaccumulate Hg is observed mainly at the root. In plants inoculated with B1 and the CS5 consortium (B1 + B2), a significant difference in the concentration of Hg in the whole plant (total, aerial, and root) is detected with respect to the control. In the aerial part of the plants subjected to the three treatments, a significant difference in the concentration of Hg with respect to the control in soils with a high concentration of Hg is also observed.

**TABLE 4:** Comparison of the concentration of Hg in the plants tested in soils with high concentration of Hg.

Treatment	Total (μg/g)	Aerial (μg/g)	Root (μg/g)
CONTROL−	0.00 ± 0.01	0.00 ± 0.01	0.00 ± 0.01
B1−	0.00 ± 0.01	0.00 ± 0.01	0.00 ± 0.01
B2−	0.00 ± 0.01	0.00 ± 0.01	0.00 ± 0.01
CS5−	0.00 ± 0.01	0.00 ± 0.01	0.00 ± 0.01
CONTROL+	10.23 ± 0.03	0.22 ± 0.02	10.01 ± 0.14
B1+	9.52 ± 0.08*	0.16 ± 0.02*	9.36 ± 0.14*
B2+	10.23 ± 0.03	0.15 ± 0.01*	10.07 ± 0.12
CS5+	7.88 ± 0.06*	0.13 ± 0.03*	7.75 ± 0.13*

*Indicates significant differences with respect to their respective controls (*p*-value ≤ 0.001).

### Principal component analysis

**FIGURE 3:**
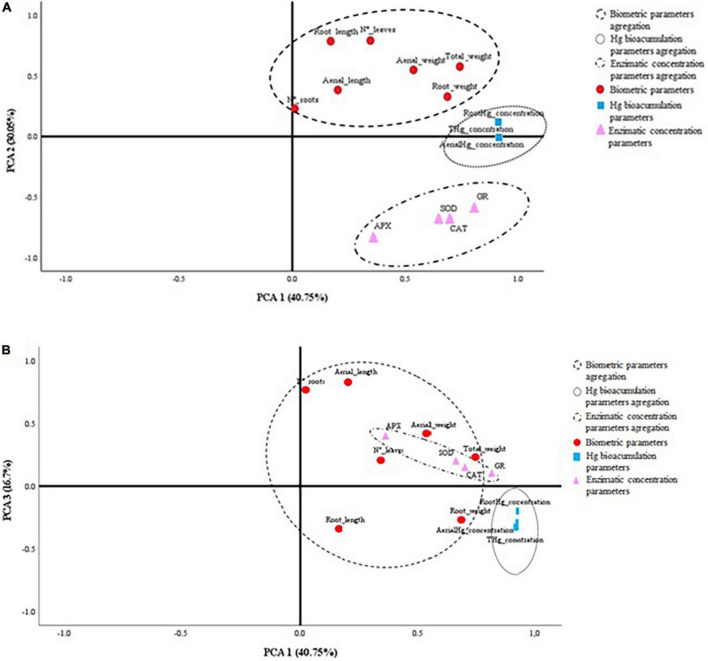
2D representation of the physiological and biometric variables of the plants according to the load factors of the PCA. **(A)** PCA1 vs. PCA2; **(B)** PCA1 vs. PCA3.

In order to discriminate the overall behavior of the plants tested on different growing matrixes with their respective inoculum, a PCA was carried out. Figure 3 shows the 2D graphs of the load factors on a rotated space of PCA1 vs. PCA2 (Figure 3A), and PCA1 vs. PCA3 (Figure 3B). The variables are segregated into three groups according to their biological behavior, namely, enzymatic activity, biometrics, and bioaccumulation of Hg. Table 5 shows that the accumulation of three factors explains the model with accumulative variance greater than 87%.

**TABLE 5:** Three main components that describe the model.

Component	Total	% variance	% acumulated
1	5.666	40.471	40.471
2	4.206	30.045	70.516
3	2.337	16.696	87.212

**FIGURE 4:**
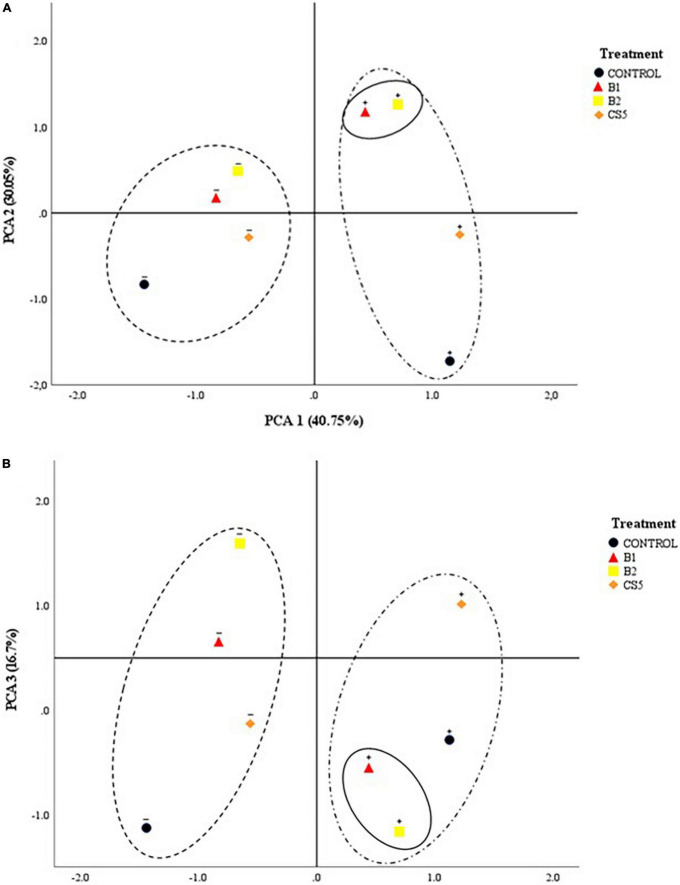
2D projection of the main components: **(A)** PCA1 vs. PCA2 and **(B)** PCA1 vs. PCA3 of the biological treatment (B1, B2, and CS5). The “+” sign on the treatment refers to “soil with a high concentration of Hg”; the “–” sign above the treatment refers to “soil without Hg.”

Figure 4 shows the 2D projected PCA model. It can be observed how the inoculum of bacteria B1 and B2 individually is segregated from the rest of the treatments. This separation corresponds to a greater effect on the decrease in enzymatic activity (ROS), as well as an increase in biometric factors. The main factor in the abscissa axis that determines the behavior of the plant turns out to be the concentration of Hg in the soil. Likewise, the main segregation factor in the ordinate axis is the treatment with an individual inoculum of PGPB B1 and B2. The phytoprotective and plant growth-promoting effects are significantly favorable in plants grown in soils with Hg when inoculated with strains B1 and B2 independently.

## Discussion

In the present study, four strains have been used whose PGP activities were tested in media with the presence of Hg vs. the absence of Hg. In the same way, their respective consortia were tested in pairs (González et al., 2021b).

The plant model of (*Lupinus albus*), as well as other legumes (Harzalli Jebara et al., 2017), has phytoextractor capacity (Zornoza et al., 2010; Rocio et al., 2013; Quiñones et al., 2021). In addition, its ability to absorb and resist the presence of heavy metals, such as Hg, is known. As well as its tolerance to high soil salinity (Rodriguez et al., 2007).

To evaluate the phytoprotective capacity of the strains against Hg, the plants were grown in two different substrates (free soil and vermiculite). Similarly, two types of soil were used to establish the comparison of the presence of Hg vs. the absence of Hg, both from the mining district of Almadén: soil with a high concentration of Hg (soil +Hg), and a control soil with a minimum concentration of Hg (soil −Hg). Vermiculite is a suitable substrate for the study of bacterial inocula in plants (Rodríguez et al., 2006; González et al., 2021a; Yuan et al., 2022) and avoids the shielding effect that a complex matrix, such as soil, can produce.

Hg induces physiological and metabolic alterations in plants, such as ROS and decreased plant growth (Çavuşoğlu et al., 2022). This article analyzes the negative influence of Hg on these variables (Figure 2). Likewise, it is known that the use of PGPB minimizes these effects (Pirzadah et al., 2018), stimulating different defense mechanisms (Loix et al., 2017).

### Antioxidative defense enzymes

Oxidative stress caused by Hg has been studied in different plant models (Cho and Park, 2000; Cargnelutti et al., 2006; Çavuşoğlu et al., 2022), observing how this heavy metal increases stress and ROS accumulation. The production of CAT, SOD, APX, and GR enzymes catalyze the degradation of H_2_0_2_, HO^–^, ^1^O_2_, and O^–^_2_. Therefore, enzymatic activity is interpreted as a protective response against ROS, whose function is induced by the effect of Hg. The increase in CAT and SOD has been studied as a marker of oxidative stress against heavy metals in plants without a bacterial inoculum (Macar et al., 2020; Çavuşoğlu et al., 2022). In the present study, it was observed that the activity of these enzymes is significantly higher in plants grown with Hg vs. without Hg (Figure 2). This effect has also been observed by other authors when confronting plants with other metals, such as cadmium (Cd) or lead (Pb) (Aras, 2012; Azimychetabi et al., 2021). Likewise, this effect has been observed in the enzymes APX and GR when facing different plant species with heavy metals (Hashem et al., 2016; Liu et al., 2018; Azimychetabi et al., 2021). Similarly, Pirzadah et al. (2018) investigate the effect of Hg on oxidative stress in plants not inoculated with PGPB, finding similar results to those described in the present work.

The effect that PGPB inoculation induces the decrease of ROS is known (Heidari and Golpayegani, 2012; Morcillo and Manzanera, 2021) in substrates contaminated by different heavy metals: Hg (Pirzadah et al., 2018), Pb (Abdelkrim et al., 2018), Cu (Fatnassi et al., 2015), Zn (Islam et al., 2014), and Cd (Azimychetabi et al., 2021; Renu et al., 2022). The PGPB species commonly used are those belonging to the genus *Bacillus* (Vardharajula et al., 2011; Moreno-Galván et al., 2020) and *Pseudomonas* (Sandhya et al., 2010). In the present study, the strains that produce a greater reduction in enzymatic activity in plants grown in the presence of Hg are B1 (*Pseudomonas baetica*) and B2 (*Pseudomonas moraviensis*) (Figures 1A–D) used both individually and in the consortium.

In the results obtained, a significant reduction in the levels of CAT (Figure 1A) and APX (Figure 1C) enzyme activities was observed. This reduction is strongly correlated with the enzymatic activity of SOD (Supplementary Table 3). The SOD enzyme catalyzes singlet oxygen into a less reactive form of oxygen (H_2_O_2_). However, H_2_O_2_ is also toxic at high concentrations and must be eliminated by conversion to H_2_O. CAT catalyzes the decomposition of H_2_O_2_ to H_2_O and O_2_. Similarly, the enzyme APX breaks down the H_2_O_2_ in H_2_O by the reducing power of ascorbic acid. Plants possess enzymes such as CAT and APX that help maintain intracellular levels of H_2_O_2_ (Gill and Tuteja, 2010). For this reason, the correlation observed between the activity of SOD enzymes against CAT and APX in plants grown in the presence of Hg acquires biological meaning and is interpreted as metabolically related phytoprotection mechanisms. The inoculum of the B1 and B2 strains induce a better response of the plant subjected to oxidative stress.

Glutathione reductase is involved in the reduction of glutathione disulfide (GSSG) to glutathione (GSH) with NADPH expenditure. GSH plays a very important role in the redox regulation of the cell cycle and in the defense mechanisms against oxidative stress (Sánchez-Fernández et al., 1997). The increase in GR in substrates with Hg (Figure 1D) corroborates what we have found and is consistent with what has been described by other authors, indicating that how Hg increases oxidative stress in the plant (Pirzadah et al., 2018). We also observed how strains B1, B2, and their CS5 consortium (Figure 1D) show significantly lower enzymatic activity of this enzyme in plants grown in soil with Hg.

### Analysis of Hg content in plant and principal component analysis

Plants of different species have been shown to accumulate Hg in different tissues, but the mechanism of absorption is unknown. To date, no membrane transporters involved in Hg root absorption have been identified. Due to the similarities between Cd and Hg, transmembrane Cd conveyors may be used (Lombi et al., 2001) for Hg input (Tiodar et al., 2021). The bioaccumulation of Hg in *Elodea nuttallii* has been analyzed, and it has been concluded that Cu transporters could be involved in the process (Regier et al., 2013). *Lupinus albus* is a known plant species accumulating Hg (Zornoza et al., 2010; Rocio et al., 2013; González et al., 2021a; Quiñones et al., 2021). Numerous metal carrier homologues have been identified in *Lupinus* roots (Tian et al., 2009). Whether these transporters could play a similar role in Hg absorption remains to be demonstrated. Quiñones et al. (2013, 2021) have used this plant species to demonstrate its ability to accumulate significant amounts of Hg in roots and nodules. This fact can induce a reduction in biomass production. This fact coincides with what has been observed in the present work. Nevertheless, plants inoculated with B1 and B2 are able to increase plant growth, even in substrates with high concentration of Hg. Likewise, there is evidence that inoculation with B1 and CS5 protects the plant against the contaminant, observing tissue concentrations of Hg significantly lower than the control (Table 4). These variables of root bioaccumulation of Hg and biometrics (total weight of the plant and root weight) present a positive correlation (Figure 3). In this same sense, the PCA segregates the behavior of plants treated with B1 and B2 in the presence of Hg. This fact leads us to think that the biological treatment with these strains in soils with a high concentration of Hg determines both the improvement of biometric variables, the reduction of the concentration of Hg in the plant, as well as the reduction of the activity of the enzymes that regulate the concentration of ROS.

The results of the present study show the capacity of phytoprotection against the accumulation of Hg and reduction of oxidative stress in *L. albus* var. orden Dorado of the strains B1 (*Pseudomonas moraviensis*) and B2 (*Pseudomonas baetica*), as well as of their respective CS5 consortium. For this reason, the convenience of using these strains for further use in phytostimulation and phytoprotection in soils contaminated with Hg is postulated.

## Conclusion

It can be extracted as a conclusion that the biological behavior of plants [biometrics, bioaccumulation of Hg and activity of catalase enzymes (CAT), superoxide dismutase (SOD), ascorbate peroxidase (APX), glutathione reductase (GR)] is significantly improved by inoculation with strains B1 (*Pseudomonas moraviensis*) and B2 (*Pseudomonas baetica*), as well as their corresponding consortium (CS5). In a particular way we can conclude as follows:

First, the bacteria B1 and CS5 exert a phytoprotective effect showing significantly lower systemic Hg concentration values and, especially, at the root. The B2 strain significantly reduces the bioabsorption of Hg in the aerial part of the plant.

Second, B1 and B2 significantly promote the plant growth of *Lupinus albus* growth. Its consortium (CS5) reduces oxidative stress, especially when the plant grows in highly contaminated soils with Hg.

In the light of the conclusions of this work, the use of strains B1 (*Pseudomonas moraviensis*) and B2 (*Pseudomonas baetica*) is postulated, as well as their consortium (CS5) as good candidates for their subsequent use phytostimulation and phytoprotection in areas contaminated with Hg.

## Data availability statement

The original contributions presented in this study are included in the article/Supplementary material, further inquiries can be directed to the corresponding authors.

## Author contributions

AP and PJ supervised the project and acquired funding for this research. All authors contributed on design the experiments, making intellectual contributions, conduct the experiments, analyze the data, writing and editing of this manuscript, and approved the submitted version.
