# Supplementary material for: Evaluation of the oxidative stress alleviation in Lupinus albus var. orden Dorado by the inoculation of four plant growth-promoting bacteria and their mixtures in mercury-polluted soils
Source: Front Microbiol. 2022 Sep 29;13:907557. doi: 10.3389/fmicb.2022.907557 (PMC9556840; doi:10.3389/fmicb.2022.907557)
Supplement: Supplementary file 1 [file Data_Sheet_1.docx]

Supplementary Material

# Supplementary information about aera

The mining district of Almadén (Ciudad Real, Spain) constitutes the largest natural concentration of mercury in the world, occupying an area of around 300km^2^. Mercury production in Almaden accounts for more than 30% (285,000 t) of the total known mercury produced in the world.

The Almadén (Ciudad Real) mining area has been exploited over the years and is considered one of the largest Hg production areas in the world. Mineral Hg deposits are found predominantly as cinnabar (HgS). Elemental mercury (Hg0) is also present and locally abundant in the atmosphere, where it can stay up to 1.7 years. Given the large size of Almadén, Hg dispersion by rivers and emissions throughout the 2000 years that the mining district has been active, it is considered one of the most contaminated areas of the planet, both due to the natural origin of Hg, and due to its anthropogenic exploitation. It’s known that Hg was extracted from this area, even before the romanization. With the roman conquest begun an intensive Hg extraction, mainly on its form of cinnabar to made tinctures, as well as metallic Hg for gold extraction.

When the exploited mine closed in 2003, other forms of land use had to be considered, such as agriculture or livestock.

The plots chosen on the present study have an acid pH (pH= 4-5). The plots were chosen by its Hg concentration characteristics and looking for two areas that were geographically near between them, in order to have the same environmental characteristics. Plot 6 was chosen to be the one which has the highest Hg interchangeable and soluble concentrations. In the same was, Plot 2 has the lowest soluble and interchangeable Hg concentrations, being low enough to be considered negligible. Having in this way, the comparative without Hg soil.

Plots characteristics:

1. Plot 2: Almadén, “Fuente del Jardinillo”. The plot is on a slope of the mountain, next to remains of the silicícola holm oak, there are scrub formations belonging to the community *Genisto hirsutae-Cistetum ladaniferi*, forming by *Cistus ladanifer*, *C. monspeliensis*, *C. crispus*, *C. salviifolius* and *C. albidus*, accompanied by other bushes and shrubs typical of the cinder formation.

- Plot 6: Almadén, mercury mine, “slope S Cerro Buitrones”. The plot is on a steep slope dominated by the species *Rumex induratus*. In areas with more fixed substrate are siliceus species such as *Cistus crispus* and *Cytisus scoparius*. And, in areas with more erodible soil, can be found therophytes such as *Rumex bucephalophorus* appear.

# Supplementary Figures and Table


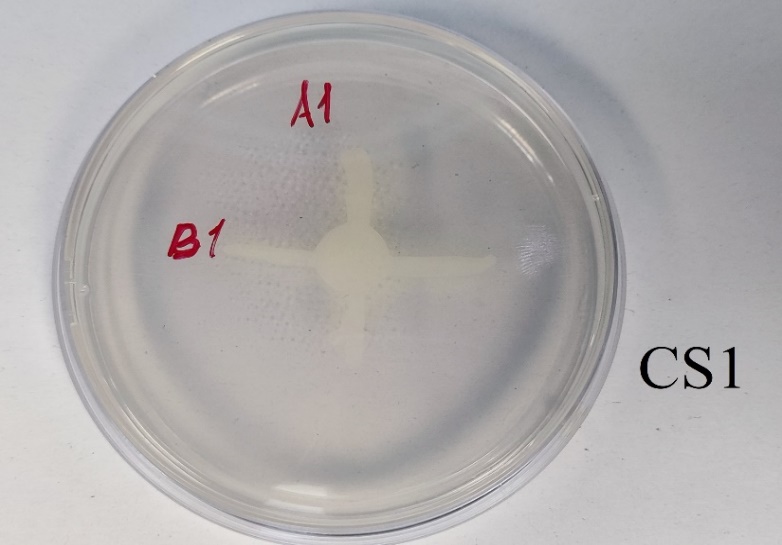

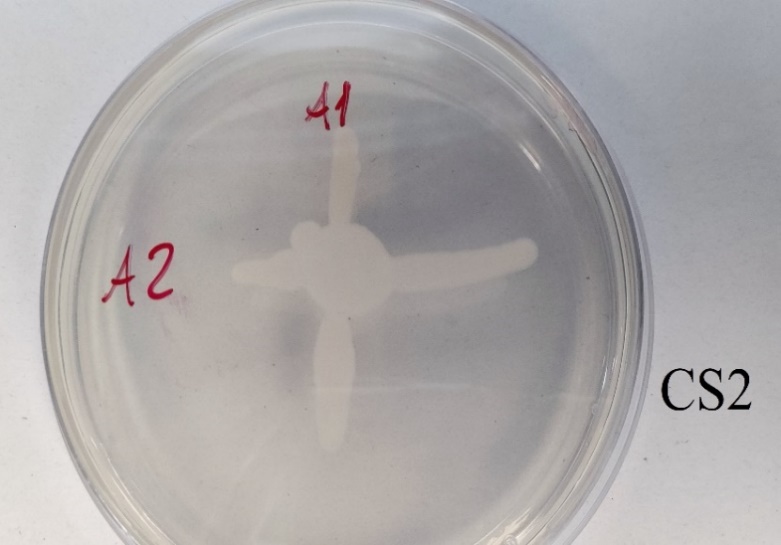


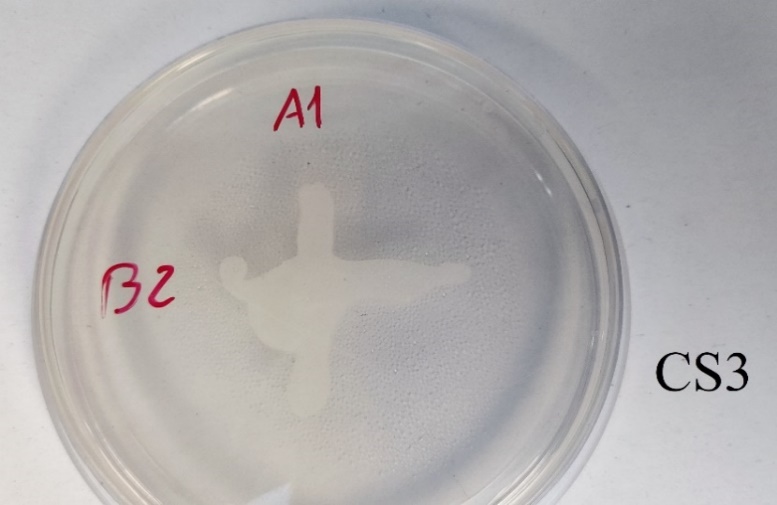

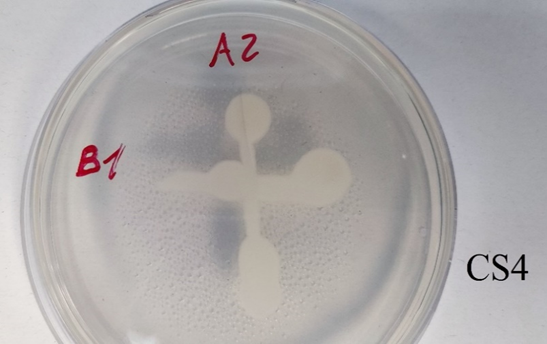


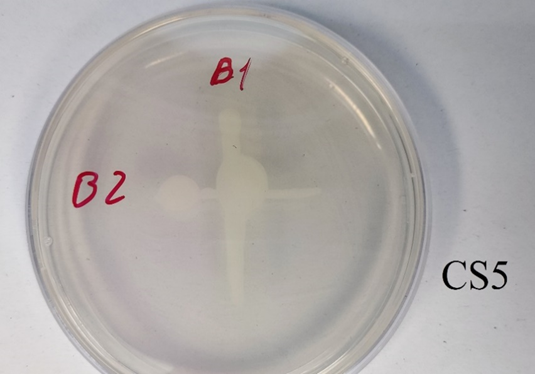

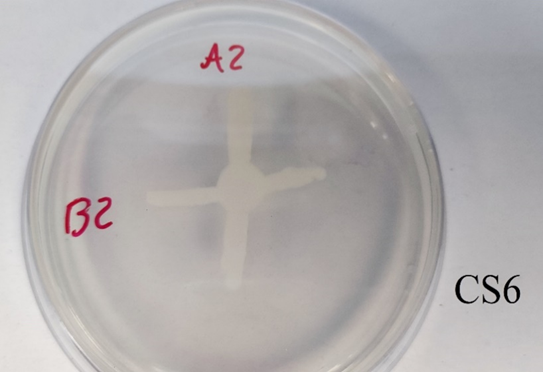


**Supplementary Figure 1.** Compatibility test for six consortia formed by the strains.

Table S1. Prameter for the calculation of the Biomercuroremediator Suitability Index (BRMSI). IAA: 3-indolacetic acid; PO_4_^3−^: Phosphate solubilization; ACCd: ACC deaminase activity; MBC: Maximum Bactericidal Concentration. 1: activity presence; 0: activity absence.

| **Strain** | **Identification** | **IAA (μg/mL)** | **PO_4_^3−^ (1/0)** | **SID (cm)** | **ACCd (1/0)** | **MBC (μg/mL)** | **BMRSI** |
| --- | --- | --- | --- | --- | --- | --- | --- |
| A1 | *Brevibacterium frigoritolerans* | 6.40 | 0 | 0 | 0 | 140 | 6.54 |
| A2 | *Bacillus toyonensis* | 6.16 | 0 | 0 | 1 | 140 | 7.30 |
| B1 | *Pseudomonas moraviensis* | 7.06 | 0 | 0 | 0 | 140 | 7.20 |
| B2 | *Pseudomonas baetica* | 6.76 | 0 | 0 | 0 | 160 | 6.92 |

**Table S2.** Biometric parameters used for the PCA analysis. Its correspond with de means of each biometric parameter for the plants growth in soil with high concentration of Hg (+) and low concentration of Hg (-).

| Treatment | Total_weight (g) | Root_weight (g) | Aerial_weight (g) | Root_length (cm) | Aerial_length (cm) | Nº_leaves | Nº_roots |
| --- | --- | --- | --- | --- | --- | --- | --- |
| CONTROL+ | 3.33 ± 0.26 | 0.77 ± 0.07 | 2.59 ± 0.22 | 18.72 ± 3.24 | 12.7 ± 0.7 | 4.2 ± 0.25 | 26.5 ± 3.85 |
| B1+ | 3.59 ± 0.18 | 0.83 ± 0.11 | 2.77 ± 0.1 | 27.55 ± 1.01 | 13.88 ± 0.4 | 5 ± 0.15 | 32.2 ± 0.29 |
| B2+ | 3.88 ± 0.22 | 1.04 ± 0.1 | 2.88 ± 0.17 | 28.37 ± 2.69 | 12.58 ± 0.38 | 4.8 ± 0.13 | 27.8 ± 4.84 |
| CS5+ | 3.77 ± 0.2 | 0.9 ± 0.06 | 2.86 ± 0.15 | 25.41 ± 2.25 | 14.23 ± 0.26 | 4.7 ± 0.21 | 40.6 ± 4.34 |
| CONTROL- | 2.76 ± 0.46 | 0.57 ± 0.18 | 2.17 ± 0.31 | 24.25 ± 2.64 | 10.28 ± 1.66 | 4.13 ± 0.81 | 30.63 ± 5.23 |
| B1- | 3.29 ± 0.3 | 0.61 ± 0.07 | 2.67 ± 0.24 | 22.58 ± 1.82 | 14.38 ± 0.65 | 4.7 ± 0.45 | 32 ± 5.04 |
| B2- | 3.5 ± 0.27 | 0.64 ± 0.07 | 2.82 ± 0.21 | 22.38 ± 2.14 | 15.54 ± 0.38 | 4.67 ± 0.25 | 42.83 ± 5.51 |
| CS5- | 3.3 ± 0.24 | 0.92 ± 0.17 | 2.82 ± 0.23 | 21.73 ± 2.24 | 13.24 ± 0.88 | 4.08 ± 0.31 | 24.75 ± 4 |

Table S3. PCA correlation matrix.

|  | Total_ weight | Root_ weight | Aerial_ weight | Root_ length | Aerial_ length | Nº_ leaves | Nº_ roots | THg_ concntration | AerialHg_ concentration | RootHg_ concentration | CAT | SOD | APX | GR |
| --- | --- | --- | --- | --- | --- | --- | --- | --- | --- | --- | --- | --- | --- | --- |
| Total_weight | 1.000 | 0.744 | 0.892 | 0.482 | 0.569 | 0.714 | 0.272 | 0.635 | 0.559 | 0.636 | 0.119 | 0.169 | -0.105 | 0.272 |
| Root_weight | 0.744 | 1.000 | 0.683 | 0.430 | 0.053 | 0.224 | -0.295 | 0.632 | 0.559 | 0.633 | 0.127 | 0.308 | -0.128 | 0.261 |
| Aerial_weight | 0.892 | 0.683 | 1.000 | 0.244 | 0.756 | 0.558 | 0.224 | 0.328 | 0.270 | 0.329 | -0.011 | 0.147 | -0.114 | 0.088 |
| Root_length | 0.482 | 0.430 | 0.244 | 1.000 | -0.067 | 0.682 | 0.154 | 0.354 | 0.221 | 0.356 | -0.486 | -0.461 | -0.641 | -0.307 |
| Aerial_length | 0.569 | 0.053 | 0.756 | -0.067 | 1.000 | 0.568 | 0.605 | -0.048 | -0.050 | -0.048 | -0.022 | 0.035 | 0.054 | 0.000 |
| Nº_leaves | 0.714 | 0.224 | 0.558 | 0.682 | 0.568 | 1.000 | 0.477 | 0.405 | 0.324 | 0.407 | -0.254 | -0.343 | -0.425 | -0.103 |
| Nº_roots | 0.272 | -0.295 | 0.224 | 0.154 | 0.605 | 0.477 | 1.000 | -0.154 | -0.172 | -0.154 | -0.011 | -0.034 | 0.192 | 0.024 |
| THg_ concntration | 0.635 | 0.632 | 0.328 | 0.354 | -0.048 | 0.405 | -0.154 | 1.000 | 0.988 | 1.000 | 0.519 | 0.395 | 0.081 | 0.656 |
| AerialHg_ concentration | 0.559 | 0.559 | 0.270 | 0.221 | -0.050 | 0.324 | -0.172 | 0.988 | 1.000 | 0.988 | 0.616 | 0.474 | 0.183 | 0.731 |
| RootHg_ concentration | 0.636 | 0.633 | 0.329 | 0.356 | -0.048 | 0.407 | -0.154 | 1.000 | 0.988 | 1.000 | 0.517 | 0.393 | 0.079 | 0.654 |
| CAT | 0.119 | 0.127 | -0.011 | -0.486 | -0.022 | -0.254 | -0.011 | 0.519 | 0.616 | 0.517 | 1.000 | 0.919 | 0.876 | 0.980 |
| SOD | 0.169 | 0.308 | 0.147 | -0.461 | 0.035 | -0.343 | -0.034 | 0.395 | 0.474 | 0.393 | 0.919 | 1.000 | 0.887 | 0.904 |
| APX | -0.105 | -0.128 | -0.114 | -0.641 | 0.054 | -0.425 | 0.192 | 0.081 | 0.183 | 0.079 | 0.876 | 0.887 | 1.000 | 0.804 |
| GR | 0.272 | 0.261 | 0.088 | -0.307 | 0.000 | -0.103 | 0.024 | 0.656 | 0.731 | 0.654 | 0.980 | 0.904 | 0.804 | 1.000 |
